# Platelet Secretome Drives Mitogenic and TGF-β Responses in Gingival Fibroblasts

**DOI:** 10.3390/biology15020143

**Published:** 2026-01-14

**Authors:** Layla Panahipour, Matilde Riberti, Xiaoyu Huang, Michael B. Fischer, Richard J. Miron, Reinhard Gruber

**Affiliations:** 1 Department of Oral Biology, University Clinic of Dentistry, Medical University of Vienna, Sensengasse 2a, 1090 Vienna, Austria; 2 Department of Blood Group Serology and Transfusion Medicine, Medical University of Vienna, Währinger Gürtel 18-20, 1090 Vienna, Austria; 3 Center for Biomedical Technology, Department for Biomedical Research, Danube University Krems, Dr.-Karl-Dorrek-Straße 30, 3500 Krems, Austria; 4 Department of Periodontology, School of Dental Medicine, University of Bern, 3010 Bern, Switzerland; 5 Austrian Cluster for Tissue Regeneration, 1200 Vienna, Austria

**Keywords:** platelet-rich fibrin (PRF), activated platelets, bioassays, mitogenic activity, IL11, CXCL8, gingival fibroblasts

## Abstract

Platelet-rich fibrin (PRF) is widely used in regenerative dentistry and oral surgery for its ability to promote tissue healing and modulate cellular responses. However, PRF contains not only platelets but also leukocytes and plasma components, complicating efforts to define the specific contribution of platelets to its biological activity. To address this, we used washed, leukocyte-depleted platelets activated with thrombin to generate platelet-released supernatant (PRS), which was applied to gingival fibroblasts. RNA sequencing identified 147 upregulated and 39 downregulated genes (|log_2_ fold change| ≥ 2, FDR < 0.001), including cytokines IL11 and CXCL8 previously associated with PRF, as well as mitosis-related genes such as centromere-associated proteins, cell division cycle proteins, kinesin-like proteins, and shugoshins, consistent with gene ontology analyses. Validation by RT-PCR and immunoassays confirmed robust upregulation of IL11 and CXCL8. Functionally, PRS activated TGF-β signaling, indicated by Smad2/3 nuclear translocation, but did not induce NF-κB signaling. These findings demonstrate that platelets are major contributors to PRF’s biological effects, independent of leukocytes and plasma, and elicit a pronounced mitogenic and TGF-β-dominant response in gingival fibroblasts. They also provide insight into the cellular mechanisms underlying PRF-mediated tissue regeneration.

## Simple Summary

Platelet-rich fibrin (PRF) is commonly used in dentistry and oral surgery to enhance wound healing and tissue regeneration. PRF is prepared from blood and contains a mixture of platelets, white blood cells, and plasma proteins, making it difficult to determine which component is primarily responsible for its healing effects. The aim of this study was to clarify the specific role of platelets. To achieve this, we isolated platelets, removed white blood cells, and collected the substances released by activated platelets. These platelet-derived factors were applied to gingival fibroblasts, which are cells essential for gum tissue repair. We found that platelet-derived factors strongly stimulated genes involved in cell growth and increased the production of molecules known to support healing. Importantly, these factors activated a key repair-related signaling pathway without triggering excessive inflammation. In conclusion, platelets play a central role in the biological effects attributed to PRF.

## 1. Introduction

Regeneration is an evolutionarily conserved process initiated by blood clot formation [1,2]. Coagulated blood fills the defect side with a fibrin-rich extracellular matrix that accumulates platelets, leukocytes, and erythrocytes [3]. Cells within the clot mount a transient immune response, which subsequently promotes fibroblast migration and proliferation, as well as neovascularization, ultimately organizing the clot into granulation tissue [1]. This fundamental biological principle of wound healing has been adapted clinically through the development of platelet-rich fibrin (PRF) [4] and its refinements [5].

PRF represents a fractionated blood clot that is depleted of erythrocytes but enriched in platelets and leukocytes within the fibrin matrix [6]. Unlike platelet-rich plasma (PRP), in which coagulation is inhibited by citrate [7,8], PRF preparation removes the liquid serum fraction, leaving a membrane containing entrapped platelets and leukocytes. This membrane is used to support the healing of soft and hard tissues in a variety of clinical contexts, including alveolar ridge preservation [9], post-extraction sites [10], and the treatment of intrabony [11] or furcation defects [12]. These clinical observations have motivated investigations into the underlying biological mechanisms of this blood-borne regenerative strategy.

Early studies characterized PRF-released growth factors [13,14,15] and assessed responses in gingival fibroblasts [15,16,17], epithelial cells [18,19,20], and peripheral blood mononuclear cells (PBMCs) [21]. These experiments confirmed the anticipated release of growth factors [22], such as transforming growth factor β (TGF-β) and platelet-derived growth factor (PDGF) [13,14,15], and their corresponding cellular effects, including upregulation of interleukin 11 (IL11) [16] and the enhanced proliferation [17,23], respectively. Collectively, these findings demonstrate the pleiotropic activity of PRF. Given PRF’s heterogeneity, consisting of platelets and leucocytes embedded in a fibrin matrix, it is difficult to attribute specific effects to individual components. In particular, the contribution of platelets—specifically the factors immediately released from their granules upon activation—to the overall cellular responses remains unclear. Previous studies have not fully dissected platelet-specific effects due to the complexity of the PRF matrix.

To address this gap, we investigated the effects of purified, activated platelets on gingival fibroblasts. Washed platelets, obtained from platelet concentrates, were suspended in culture medium and exposed to thrombin to trigger rapid granule release [24,25]. After centrifugation, the platelet-released supernatant (PRS), also known as the releasate [26,27], was separated from the remaining pellet. PRS, therefore, represents the factors released within 30 min of platelet activation, a process termed platelet exocytosis [28]. Using RNA sequencing, we captured the full genomic response of gingival fibroblasts exposed to PRS, analog to prior studies with PRF lysates and serum [29]. Importantly, this approach isolates platelet-specific effects, that are independent of leukocytes and plasma components.

Here, we present evidence that PRS elicits a robust mitogenic and TGF-β-dominant response in gingival fibroblasts, highlighting the critical role of platelets in the biological activity of PRF.

## 2. Materials and Methods

### 2.1. Isolation of Platelet Concentrates

Platelet concentrates from volunteer donors were prepared by single-donor aphaeresis (Trima Accel automated blood collection system, Terumo BCT, Lakewood, CO, USA) using acid citrate dextrose (ACD-A; 22 g/L sodium citrate, 24.5 g/L glucose monohydrate and 8 g/L citric acid monohydrate) as anticoagulant at a ratio of 1:10. Platelet concentrates were routinely subjected to a leukocyte reduction system (LRS) during the process of apheresis, where fewer than 3 × 10^3^ mononucleated cells/mL remained in the product as per standard blood banking regulations; thus the leucocyte depletion factor is approximately three decimal powers [30]. Platelet concentrates were collected and processed at the Clinic for Transfusion Medicine and Cellular Therapy of the Medical University of Vienna (MUW). Aliquots (5 mL) were aseptically withdrawn for quality control purposes, and the remaining material was used to prepare PRS. This study was conducted in accordance with the Austrian Hospital Act (Krankenanstaltengesetz, KAG 1982), the federal framework law regulating hospital-based clinical activities in Austria, which ensures ethical oversight and protection of patients in compliance with national legislation and was approved by the ethics commission of the MUW [31] and the University of Continuing Education Krems. All donors gave written informed consent before participating in the study. Since the donors stayed anonymous, we can only state that they were healthy adults qualified for blood donation.

### 2.2. Preparation of Platelet-Released Supernatants (PRS)

For each of the three independent preparations, aliquots from 2 to 3 donors were pooled, and three mL platelet concentrates were obtained, washed with an excessive volume of buffered saline, and centrifuged at 1400× *g* for 10 min. Pellets corresponding to 3 × 10^9^ platelets/mL were resuspended in 3 mL of serum-free medium, which consisted of Dulbecco’s Modified Eagle Medium (DMEM; Sigma Aldrich, St. Louis, MO, USA) supplemented with antibiotics (Invitrogen Corporation, Carlsbad, CA, USA). Platelet activation was induced by adding 1 U/mL of human thrombin (Sigma, St. Louis, MO, USA) for 30 min at room temperature. After centrifugation, the three independent PRS preparations were stored in aliquots at −20 °C prior to fibroblast exposure.

### 2.3. Human Gingival Fibroblast Isolation and Experimental Setting

Human gingival fibroblasts were prepared by explant culture of gingiva obtained through the removal of wisdom teeth from healthy donors who provided informed consent. The Ethical Committee of the Medical University of Vienna approved the protocol (EK Nr. 631/2007). Cells were cultured in a humidified atmosphere at 37 °C in a growth medium consisting of DMEM, 10% fetal calf serum (FCS), and 1% antibiotics (Invitrogen Corporation, Carlsbad, CA, USA). We generated three independent fibroblast preparations pooled from different donors. Gingival fibroblasts of low passage were seeded at 3 × 10^4^/cm^2^ the day before being exposed to 30% PRS corresponding to 3 × 10^8^ platelets/mL in serum-free medium for an additional 16 h, followed by the isolation of RNA and storage of the supernatant for immunoassay. Recombinant human TGF-β1, IL1 β, and TNFα (ProSpec-Tany TechnoGene Ltd., Rehovot, Israel) at 10 ng/mL and the TGF-β receptor type I kinase inhibitor SB431542 (Calbiochem, Merck, Billerica, MA, USA) at 10 µM were used.

### 2.4. Total RNA Isolation, RNA Sequencing, and Data Analysis

Total RNA from three independent experiments was isolated with the GeneMATRIX Universal RNA purification kit with DNAse digestion (EUR_X_, Gdańsk, Poland). Sequencing libraries from total RNA were prepared at the Core Facility Genomics, Medical University of Vienna, using the QuantSeq 3′ FWD protocol version 2 with unique dual indices (Lexogen GmbH, Vienna, Austria). The number of PCR cycles for library preparation was set to fifteen. Library quality control was performed using a Bioanalyzer 2100 (Agilent Technologies, Santa Clara, CA, USA) with a High Sensitivity DNA Kit to verify insert size, and library concentrations were measured using the Qubit dsDNA HS Assay (Invitrogen, Waltham, MA, USA). Libraries were pooled and sequenced on a P2 flow cell using a NextSeq 2000 platform (Illumina, San Diego, CA, USA) in single-end mode with a read length of 75 bp. An average of approximately 7 million sequencing reads was obtained per sample. FASTQ files were generated using the Illumina bcl2fastq command-line tool (v2.19.1.403), followed by demultiplexing with the Lexogen idemux software. Read preprocessing was carried out using cutadapt version 2.8 to remove poly(A) tails, eliminate reads containing ambiguous nucleotides, and trim low-quality bases (Phred score < 30) from the 3′ ends [32]. Following trimming and filtering, an average of approximately 5 million reads per sample remained. The processed reads in FASTQ format were aligned to the human reference genome (GRCh38) using GENCODE release 29 annotations with the STAR aligner (version 2.6.1a) operating in two-pass mode [33]. STAR was used to generate raw gene-level read counts. Read counts were normalized using the median-of-ratios method implemented in DESeq2 (version 1.22.2) [34], and batch effects were accounted for by including the donor as a covariate in the analysis. Log_2_ fold changes were shrinkage-adjusted using lfcShrink with the apeglm method, yielding stable estimates while preserving gene ranking. Multiple-testing correction was applied using the Benjamini–Hochberg false discovery rate (FDR) procedure. Biological replicates were derived from independent donors, ensuring that observed differences reflect true biological variation. Biological replicates in our study were derived from independent donors (fibroblasts and PRS), ensuring that they reflect true biological variability rather than technical replication. The sequencing data supporting this study are available in the Gene Expression Omnibus (GEO) under accession number GSE311711 (released 28 November 2025).

### 2.5. Volcano Plot, Protein–Protein Interactions, and Gene Set Enrichment Analysis

Volcano plots were generated using VolcaNoseR, a web-based visualization tool [35]. Genes were classified as up-or downregulated for downstream analyses based on a minimum absolute log_2_ fold change of 3 and a significance threshold of −log_10_ (*p*-value) ≥ 2.0 [36]. Heat map analysis was performed with Morpheus (https://software.broadinstitute.org/morpheus, accessed on 25 November 2025). The g: Profiler was used as a functional enrichment analysis tool that integrates multiple databases, including Gene Ontology [35].

### 2.6. Reverse Transcription Quantitative Real-Time PCR (RT-qPCR) and Immunoassay

Complementary DNA (cDNA) was synthesized from total RNA by reverse transcription using reagents from LabQ (Labconsulting, Vienna, Austria). Quantitative polymerase chain reaction (PCR) was subsequently performed using LabQ reagents on a CFX Connect™ Real-Time PCR Detection System (Bio-Rad Laboratories, Hercules, CA, USA). Primer sequences used in this study are provided in Supplementary Table S6. The amount of each specific mRNA was normalized to the housekeeping gene GAPDH using the ΔΔCt method. RT-qPCR data were normalized to the unstimulated control, which was set to 1.0 in all analyses. For the immunoassay, IL11 and CXCL8 protein levels in the supernatant were measured according to the manufacturer’s instructions (R&D Systems, Minneapolis, MN, USA).

### 2.7. Immunofluorescent Analysis

Cells seeded onto Millicell EZ slides (Merck KGaA, Darmstadt, Germany) were serum-starved overnight and then exposed to 30% PRS for 1 h. After 4% paraformaldehyde fixation and blocking with 1% bovine serum albumin (BSA, Sigma Aldrich, St. Louis, MO, USA), cells were incubated with Smad2/3 antibody (D7G7 XP^®^ rabbit mAb #CS-8685, Cell Signaling, Danvers, MA, USA) and NF-κB p65 (D14E12. XP^®^ Rabbit mAb #CS-8242, Cell Signaling Technology, Danvers, MA, USA) overnight at 4 °C. Subsequently, Alexa Fluor 488-conjugated secondary antibody (#CS-4412, Cell Signaling Technology, Danvers, MA, USA) was used for one hour at room temperature. Finally, cells were washed and mounted onto glass slides. Images were captured using a fluorescent microscope (Axio Imager M2, Carl Zeiss AG, Oberkochen, Germany).

### 2.8. Statistical Analysis

The experiments were repeated at least four times. Statistical analysis was performed using a ratio-paired *t*-test in Prism v9 (GraphPad Software, La Jolla, CA, USA). *p*-values are reported.

## 3. Results

### 3.1. Principal Component Analysis (PCA) and Heat Map of Gene Expression Changes

**Figure 1:**
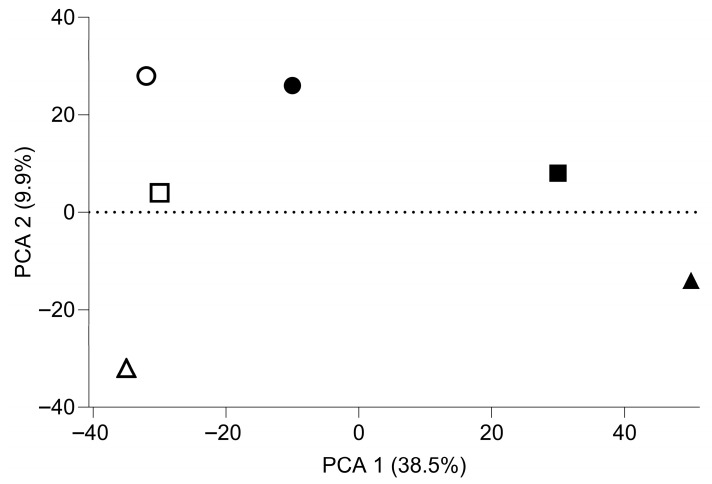
Principal component analysis of differentially expressed genes in PRS-treated gingival fibroblasts. Normalized log CPM values were used. Control samples are shown in white and PRS-treated samples in black; symbols represent three independent experiments. White denotes control; black denotes PRS.

**Figure 2:**
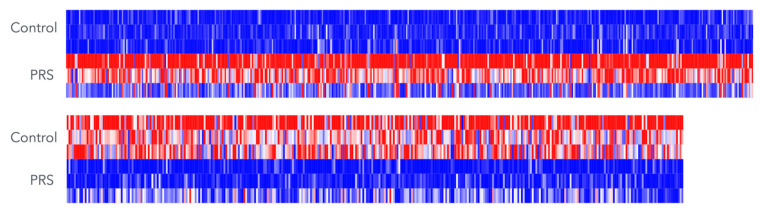
Heat map of differentially expressed genes in PRS-treated gingival fibroblasts. Genes with an FDR-adjusted *p*-value < 0.05 are shown, integrating data from three independent cell and PRS preparations. Red indicates upregulated genes and blue indicates downregulated genes, with color intensity reflecting expression levels.

To obtain an overview of gene expression changes in gingival fibroblasts exposed to PRS, a principal component analysis was performed. Figure 1 shows a clear shift caused by PRS exposure, particularly in PCA1, whereas PCA2 distinguishes the three independent experiments. Thus, there is a consistent response of gingival fibroblasts to PRS in all independent experiments, and it becomes evident that one of the three experiments causes only a moderate shift relative to the substantial changes in the other two experiments. The weaker expression change in one of the three experiments is also apparent in the heat map based on the significantly regulated genes, with an FDR-adjusted *p*-value < 0.05 (Figure 2). Thus, the heat map confirms the PCA, indicating that two out of the three independent experiments account for the majority of observations.

### 3.2. Volcano Analysis of Gene Expression Changes by PRS-Exposed Gingival Fibroblasts

**Figure 3:**
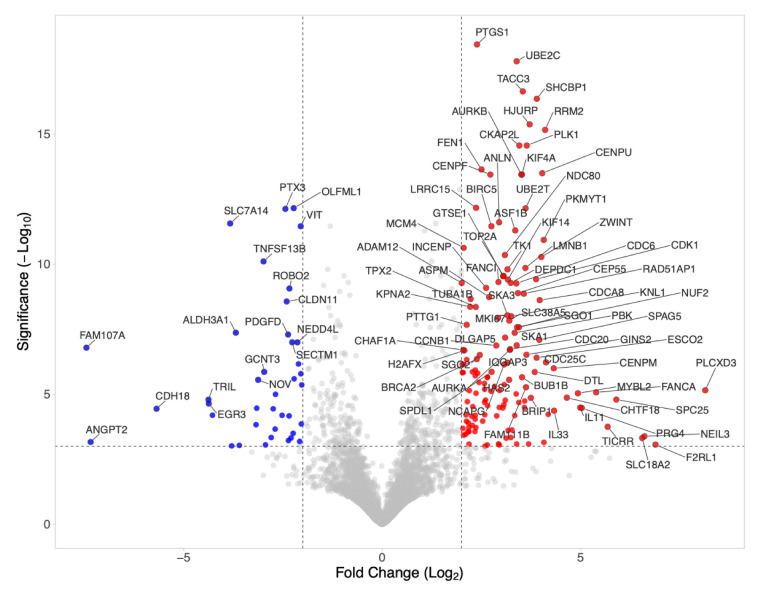
Volcano plot of differentially expressed genes in PRS-treated gingival fibroblasts. Upregulated genes are shown in red and downregulated genes in blue. Annotated points represent the 100 genes with the largest Manhattan distance from the origin and exceeding the dashed significance thresholds. Differential expression was defined as |log_2_ fold change| ≥ 2 with an FDR-adjusted *p*-value < 0.001.

Next, we performed a visualization to illustrate the direction and magnitude of gene-expression changes. The volcano plot combines the DEGs with thresholds of |log_2_ fold change| ≥ 2 and FDR-adjusted *p*-value < 0.001. We identified 147 upregulated and 39 downregulated genes in gingival fibroblasts exposed to PRS under these stringent criteria (Supplementary Tables S1–S3). Figure 3 shows the top 100 genes based on the Manhattan distance approach. The volcano plot identifies TGF-β-regulated genes, such as IL11 and PRG4, which are upregulated, and PTX3 among the downregulated genes. Among the 147 upregulated genes are also genes characteristic for cell proliferation such as centromere-associated proteins (CENPA, E, F, M, U, W), cell division cycle proteins (CDCA2, A3, A5, A8, 6, 20, 25A, 25C, 45), kinesin-like proteins (KIF11, 14, 18A, 18B, 20A, 23, 4A, C1), and shugoshin 1 and 2 as well as the inflammatory chemokine CXCL8. The boost in proliferation genes supports previous observations on the substantial impact of PRF on mesenchymal cell proliferation [24,25].

### 3.3. G:Profiler Analysis of Gene Expression Changes in Gingival Fibroblasts with PRS

**Figure 4:**
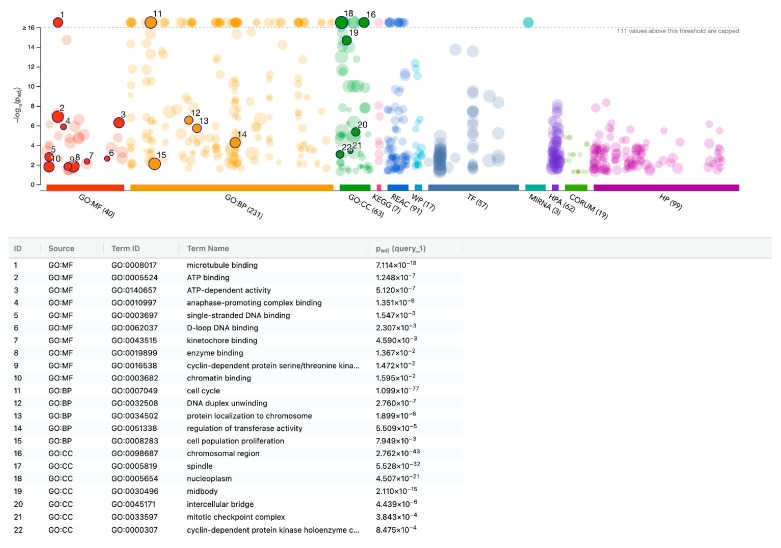
gProfiler analysis of up-regulated genes in gingival fibroblasts with PRS. Functional enrichment analysis (over-representation analysis (ORA) or gene set enrichment analysis) was performed using the g: Profiler online tool. The overall impression is that genes are enriched in cell proliferation and all related aspects.

We further performed a functional enrichment analysis, which identified PRS-mediated mitogenic activity as the primary cellular response (Figure 4, Supplementary Table S4). The mitogenic activity is exemplified by the high significant enrichment (*p* = 10^−77^) for GO:BP cell cycle GO:0007049 with the following genes being enriched: ANLN, ASPM, AURKA, AURKB, BIRC5, BLM, BRCA2, BRIP1, BUB1, BUB1B, CCNA2, CCNB1, CCNB2, CCNF, CDC20, CDC25A, CDC25C, CDC45, CDC6, CDCA2, CDCA3, CDCA5, CDCA8, CDK1, CDKN3, CENPA, CENPE, CENPF, CENPM, CENPU, CENPW, CEP55, CHAF1A, CHTF18, CKS1B, CLSPN, DBF4, DDIAS, DLGAP5, DTL, E2F1, ERCC6L, ESCO2, EXO1, FANCA, FANCD2, FANCI, FBXO5, FEN1, GTSE1, HELLS, HJURP, INCENP, IQGAP3, KIF11, KIF14, KIF18A, KIF18B, KIF20A, KIF23, KIF4A, KIFC1, KNL1, KNSTRN, MAD2L1, MCM4, MCM8, MELK, MKI67, MYBL2, NCAPG, NDC80, NUF2, NUSAP1, PBK, PIMREG, PKMYT1, PLK1, PRC1, PSRC1, PTTG1, RAD51AP1, REEP4, RRM2, SGO1, SGO2, SKA1, SKA3, SPAG5, SPC24, SPC25, SPDL1, STIL, SUV39H1, TACC3, TICRR, TK1, TOP2A, TPX2, TRIP13, TTK, TUBA1B, UBE2C, WDHD1, WD37, R76, ZWINT.

**Figure 5:**
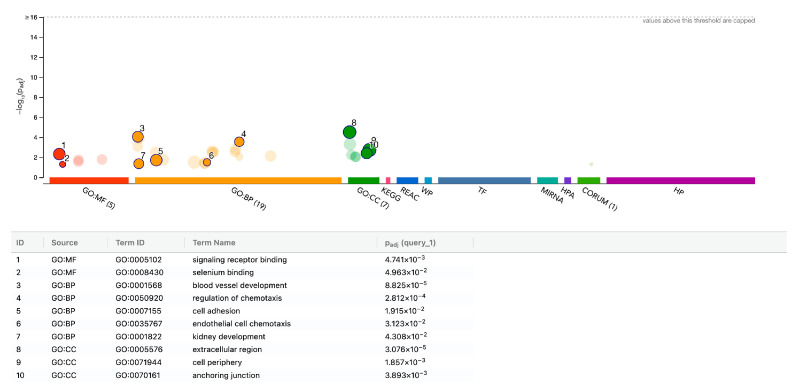
gProfiler analysis of down-regulated genes in gingival fibroblasts exposed to PRS. Functional enrichment analysis (over-representation analysis (ORA) or gene set enrichment analysis) was performed using the g: Profiler online tool. This enrichment is less consistent and lower than that for the upregulated genes.

Additionally, the transcription factors implicated in the expression changes, including HMGXB3, NFYA, NFYB, SP2, and YB-1, are associated with cell proliferation. The same is true for the hsa-miR-193b-3p, hsa-miR-215-5p, and hsa-miR-192-5p. The functional annotation of the downregulated genes is less pronounced, as indicated in Figure 5 and Supplementary Table S5. When being more inclusive with an adjusted *p* < 0.05, we identified upregulated genes within specific GO categories, including GO:0030020 extracellular matrix structural constituent conferring tensile strength (COL4A1, COL5A3, COL7A1; 3 of 28 genes), GO:0005125 cytokine activity (IL11, IL32, IL33, CXCL1, CXCL6, CXCL8, CXCL13; 7 of 234 genes), and GO:0006956 complement activation (SERPING1, C2, CFD; 3 of 60 genes), suggesting a more complex cellular response that is not restricted to mitogenesis but also includes matrix production, cytokine and chemokine expression, and complement system activity.

### 3.4. IL11 and PRG4 Expression and Smad2/3 Staining in Gingival Fibroblasts Exposed to PRS

**Figure 6:**
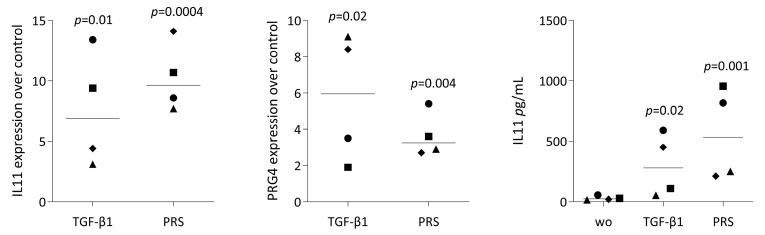
Gene expression of gingival fibroblasts exposed to PRS. Gingival fibroblasts were treated with 30% PRS. Recombinant TGF-β1 served as a positive control. ‘Without’ (wo) indicates serum-free medium alone. The expression levels of interleukin 11 (IL11) and proteoglycan 4 (PRG4) were measured using quantitative RT-PCR. IL11 protein levels were determined by immunoassay. Statistical analysis was conducted on data from four independent experiments, with different symbols representing each, using a ratio-paired *t*-test with untreated cells as controls.

**Figure 7:**
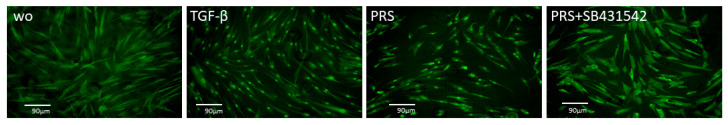
Nuclear translocation of Smad2/3 in response to PRS. Gingival fibroblasts were exposed to 30% PRS with and without the TGF-β receptor type I kinase inhibitor SB431542. Recombinant TGF-β1 served as a positive control. ‘Without (wo)’ refers to serum-free medium alone and functions as the negative control in our experiments. The positive signals induced by PRS are indicated by nuclear staining of Smad2/3, which is blocked by SB431542.

Next, we selected a small panel of genes to verify the RNA-seq analysis and to strengthen the concept that, at least in vitro, the cellular responses to PRS are strongly related to TGF-β activity. PRS exposure of gingival fibroblasts caused the expected increase in IL11 and PRG4 expression [16] (Figure 6). Consistent with the expression of TGF-β target genes, PRS induced smad2/3 nuclear translocation in gingival fibroblasts, which was sensitive to the TGF-β receptor type I kinase inhibitor SB431542 (Figure 7). Together, these findings support the concept that platelets are a rich source of TGF-β activity.

### 3.5. CXCL8 and IL33 Expression and p65 Staining in Gingival Fibroblasts Exposed to PRS

**Figure 8:**
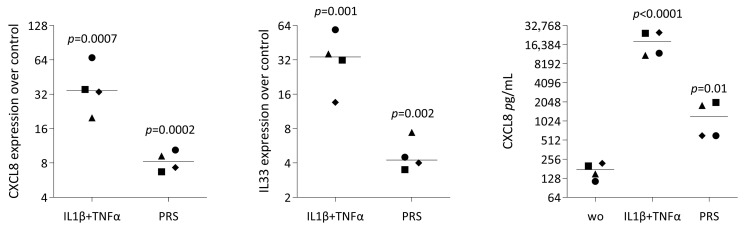
RT-PCR analysis of differentially expressed genes in gingival fibroblasts treated with PRS. Gingival fibroblasts were seeded onto a tissue culture-treated surface and, the following day, exposed to 30% PRS overnight in serum-free DMEM, followed by RT-PCR analysis. Gene expression changes were calculated using the ∆∆CT method, and results are expressed as x-fold increases relative to unstimulated cells, which have an expression level of 1. The statistics are based on a ratio-paired *t*-test, and the data points represent four independent experiments, each with a different symbol.

**Figure 9:**
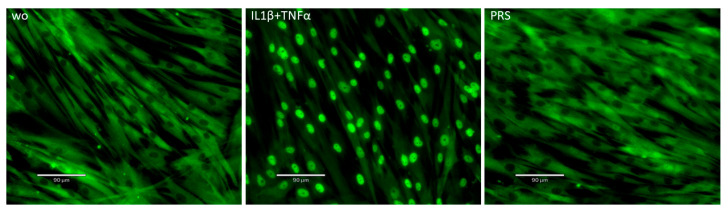
Nuclear translocation of NF-κB p65 in response to PRS. Gingival fibroblasts were exposed to 30% PRS, with IL1β and TNFα serving as positive controls. ‘Without (wo)’ refers to the serum-free medium alone and acts as the negative control in our experiments. Nuclear staining shows positive signals induced by IL1β and TNFα, whereas no signal is observed with PRS.

Finally, we observed increased expression of chemokines and cytokines, notably CXCL8 and IL33, respectively. Consistent with previous observations obtained with PRF lysates and PRF serum [29], RT-qPCR and immunoassay analyses confirmed that PRS moderately increased CXCL8 and IL33 expression, although to a significantly lesser extent than the classical inflammatory stimuli IL-1β and TNFα (Figure 8). Moreover, whereas IL-1β and TNFα robustly induced nuclear translocation of NF-κB p65 in gingival fibroblasts, PRS did not (Figure 9). Collectively, these findings indicate that PRS does not activate NF-κB signaling and therefore does not trigger a strong inflammatory response.

## 4. Discussion

Platelet-rich fibrin (PRF) is a heterogeneous matrix composed of platelets, leukocytes, and plasma-derived components that are applied clinically in an autologous setting [5]. Because PRF-based bioassays inherently represent leukocytes and plasma constituents, they do not exclusively reflect platelet-mediated effects on target cells. This limitation prompted us to investigate the specific contribution of platelets, independent of leukocytes and plasma constituents, to cellular responses in vitro. To this end, we generated a platelet releasate supernatant (PRS) by activating purified, washed platelets with thrombin [24,25], also referred to as platelet releasate [26,27]. We have shown recently that this PRS exhibits pronounced mitogenic activity in cells of the mesenchymal lineage [24,25]. Building on our previous findings and the ongoing debate regarding the specific role of platelets in PRF, we here delineate the distinct molecular and cellular signatures of fibroblasts stimulated with PRS.

We observed inter-experimental variation, as indicated by principal component analysis and heatmap comparisons. These batch effects were controlled, yielding stable estimates while preserving gene-ranking fidelity. Under these conditions, RNA-seq analysis revealed that PRS induced substantial transcriptional changes, with 147 genes upregulated and 39 genes downregulated. Functional annotation indicated (i) a marked enhancement of cell proliferation and (ii) activation of TGF-β signaling pathways, findings consistent with established platelet-derived signaling mechanisms.

The potent mitogenic activity of PRS previously reported [24,25] is concordant with the RNA-seq data, which revealed strong enrichment of mitosis-associated genes. Notably, six centromere-associated proteins were coordinately upregulated, highlighting a robust proliferative program induced by PRS [37]. In addition, PRS increased the expression of a broad spectrum of mitosis-related genes, including nine cell division cycle–associated proteins, eight kinesin family members [38], and two shugoshins [39,40]. Further upregulated mitotic regulators included polo-like kinases [41], DNA replication complex GINS proteins [42], condensin complex subunits [43], and cyclins A2, B1, and B2 [44]. PRS also enhanced the expression of DNA replication licensing factors [45] and spindle-associated Ndc80 complex components, which are essential for kinetochore–microtubule attachment and force generation during mitosis [46]. In addition, the proto-oncogene-like transcription factors MYBL1 and MYBL2 [47], and the kinetochore proteins Spc24 and Spc25 [48] were upregulated, further supporting enhanced proliferative signaling.

Beyond classical cell cycle regulators, PRS induced expression of metallothioneins MT1A and MT2A, genes transcriptionally regulated by heavy metals [49], likely reflecting zinc release from activated platelets [50,51]. Thus, in addition to established mitogens such as PDGF and FGF2 [22], the fibroblast response to PRS likely reflects the full spectrum of platelet-derived bioactive components, including zinc and bioactive lipids [52], extending beyond traditional growth factor signaling.

The growth factor TGF-β regulates IL11, PRG4, and PTX3 [16], all of which are expressed differently following stimulation with PRF lysates and, as shown here, with PRS [29]. Notably, IL11 is not a typical inflammatory cytokine and has overlapping physiological roles, making it a potential therapeutic target [53,54]. Additionally, IL33, an alarmin released passively during cell necrosis [55], and stanniocalcin-1 (STC1), a hormone with multiple functions [56], are induced by PRF serum and lysates [29]. Despite these similarities, the set of genes commonly regulated in gingival fibroblasts by PRS and PRF lysates or by serum remains relatively small [29]. Importantly, PRF serum activity does not fully mirror PRS effects, since fibroblast responses cannot be explained by platelet activation alone. In fact, PRS shows significantly higher mitogenic activity than PRF lysates or serum, likely because plasma components capture platelet-derived mitogens such as PDGF and FGF2 [57].

PRS did not induce appreciable expression of inflammatory chemokines nor nuclear translocation of p65. In contrast, PRF lysates—and to a lesser extent PRF serum—upregulated a panel of chemokines CXCL1, CXCL2, CXCL3, CXCL5, CXCL6, CXCL8, CCL2, and CCL7, accompanied by activation of NF-κB signaling in gingival fibroblasts [29]. The CXCL8 induction observed here does not necessarily require NF-κB, as it can also be triggered by connective tissue growth factor [58] or platelet-derived lipids such as lysophosphatidic acid and sphingosine-1-phosphate [59,60]. Thus, while RNA-seq data clearly demonstrate distinct fibroblast responses to PRS versus PRF, the molecular mechanisms underlying these differential effects remain incompletely understood. Importantly, PRF should not be considered synonymous with platelet activity alone.

Platelets used for PRS preparation were isolated from single-donor apheresis platelet concentrates routinely used to treat thrombocytopenia and bleeding disorders. These preparations contain negligible leukocyte contamination and achieve approximately, a fivefold enrichment of platelets relative to whole blood [61]. Accordingly, conclusions regarding platelet-derived effects can be interpreted with minimal concern for leukocyte contribution [30]. Moreover, platelet activation occurs within minutes, and PRS reflects bioactive molecules stored in platelet granules and released within 30 min following thrombin exposure [62], a process known as platelet exocytosis [28]. In contrast, leukocyte-derived protein production requires transcriptional activation and typically exceeds one hour [63], and leukocytes are not fully activated by thrombin within 30 min [64]. Thus, PRS predominantly represents the immediate secretory output of activated platelets. Nevertheless, PRS only partially recapitulates platelet activity within a blood clot or PRF, as platelet remnants remain in the pellet or become embedded within the fibrin matrix [65]. Future studies should address the bioactivity of these remnants and the contribution of extracellular vesicles [66].

Comparative analyses of PRF lysates revealed both overlapping and distinct gene-expression patterns. Venn analysis identified 99 commonly upregulated genes between PRF lysates [29] and PRS, including chemokine- and integrin-related genes (SERPINE1, IL11, CXCL1, CXCL8, PLAUR, CXCL6, IL33, ANPEP, G0S2) and genes involved in collagen trimerization (COL4A1, COL5A3, PLOD2, SUV39H2). However, the majority of upregulated genes were unique: 1144 for PRF lysates and 382 for PRS. PRF lysate-specific genes were enriched in pathways related to rRNA processing, TNF signaling, mitochondrial protein import, and overall cellular complexity, whereas PRS-specific genes predominantly clustered in mitosis, amino acid biosynthesis, and CXCR chemokine receptor binding. These findings suggest that PRS accounts for only a subset of PRF lysate activity, with leukocyte lysates and plasma components accounting for much of the remaining complexity. Similarly, comparisons with PPP lysates revealed substantial overlap in gene expression, indicating that PPP retains significant platelet-derived activity [67]. Nonetheless, PPP lysates induced a more complex transcriptional response than PRS, reinforcing the concept that PRF and PPP effects extend beyond platelet activation.

This study has limitations but also provides a foundation for future research. PRS represents a rapidly released platelet secretome composed of multiple bioactive molecules, and RNA-seq, therefore, serves as a functional bioassay rather than a tool to identify single agonists driving fibroblast responses. Importantly, these findings underscore that the clinical effects of PRF cannot be attributed solely to platelet content, and that preparation protocols aimed exclusively at platelet enrichment may overlook the biological contributions of plasma and leukocyte-derived components.

## 5. Conclusions

PRS, derived from purified and activated platelets, drives a robust mitogenic and TGF-β-dominant response in gingival fibroblasts, independent of plasma components and leukocytes. While PRF accounts for part of the proliferative activity, the broader effects of PRF likely reflect additional contributions from leukocytes, plasma constituents, platelet remnants, and extracellular vesicles. These findings highlight the pivotal role of platelet-derived factors in tissue regeneration and provide a framework for dissecting the distinct contributions of PRF components in future studies.

## Data Availability

The original contributions presented in this study are included in the article/Supplementary Material. The sequencing data supporting this study are available in the Gene Expression Omnibus (GEO) under accession number GSE311711 (released 28 November 2025).

## Supplementary Materials

The following supporting information can be downloaded at https://www.mdpi.com/article/10.3390/biology15020143/s1: Table S1: Paired_lfcShrink_apeglm_DESeq2results_GeneSymbols_GeneTypes_forVolcano. Table S2: Paired_lfcShrink_apeglm_DESeq2results_GeneSymbols_GeneTypes_padj005_up. Table S3: Paired_lfcShrink_apeglm_DESeq2results_GeneSymbols_GeneTypes_padj005_down. Table S4: Upregulated GProfiler. Table S5: Downregulated GProfiler. Table S6: Primers.
