# Supplementary material for: Platelet Secretome Drives Mitogenic and TGF-β Responses in Gingival Fibroblasts
Source: Biology (Basel). 2026 Jan 14;15(2):143. doi: 10.3390/biology15020143 (PMC12838376; doi:10.3390/biology15020143)
Supplement: Supplementary file 1 [file biology-15-00143-s001.zip › Table S6 Primers.pdf]

The primer sequences.

Genes	Forward Sequence	Reverse Sequence
IL11	CGAGCGGACCTACTGTCCTA	GCCCAGTCAAGTGTCAGGTG
PRG4	CAGTTGCAGGTGGCATCTC	TCGTGATTCAGCAAGTTTCATC
CXCL8	AACTTCTCCACAACCCTCTG	TTGGCAGCCTTCCTGATTTC
IL33	GTGACGGTGTTGATGGTAAGAT	AGCTCCACAGAGTGTTCCTTG
GAPDH	AGCCACATCGCTCAGACAC	GCCCAATACGACCAAATCC
